# Intraoperative Use of Topical Retropharyngeal Steroids for Dysphagia after Anterior Cervical Fusion: A Systematic Review and Meta-Analysis

**DOI:** 10.1155/2021/7115254

**Published:** 2021-12-31

**Authors:** Hang Yu, Hui Dong, Binjia Ruan, Xiaohang Xu, Yongxiang Wang

**Affiliations:** ^1^Clinical Medical College of Yangzhou University, Yangzhou, China; ^2^Northern Jiangsu People's Hospital Affiliated to Yangzhou University, Yangzhou, China; ^3^The Yangzhou School of Clinical Medicine of Dalian Medical University, Yangzhou, China

## Abstract

**Purpose:**

The anterior cervical approach is commonly used clinically for cervical spondylosis, but it also results in frequent postoperative dysphagia, which can increase the risk of complications and poor treatment satisfaction in severe cases. Intraoperative local application of retropharyngeal steroids has an impact on reducing the occurrence and severity of dysphagia; however, the results of current studies vary. The meta-analysis of this randomized trial was to evaluate the effectiveness and safety of intraoperative topical retropharyngeal steroids for the control of dysphagia after anterior cervical spine surgery.

**Methods:**

Two authors searched electronic databases such as PubMed, MEDLINE, EMBASE, Cochrane Library, and Google Scholar, respectively. The search terms were “Dysphagia,” “Steroids,” “Anterior Cervical Discectomy and Fusion,” etc. A random effects model was used to conduct a meta-analysis based on deviance information criteria.

**Results:**

A total of 8 studies were included in this meta-analysis after screening of 792 studies. Bazaz scores were not significantly different in the steroid group at one day postoperatively (*P* = 0.38), and dysphagia was significantly improved at 14 days postoperatively (95% CI: 0.15 to 0.64; *P* = 0.002). PSTSI was significantly improved one day (*P* = 0.03) and 14 days after surgery (*P* < 0.0001). VAS scores were all lower versus controls (*P* < 0.001).

**Conclusion:**

Perioperative local retropharyngeal steroid administration as an adjunct to anterior cervical spine surgery reduces the incidence and severity of dysphagia compared with placebo control. However, future high-quality randomized controlled studies could incorporate nonsubjective dysphagia measures and long-term follow-up on the occurrence of associated complications or other side effects.

## 1. Introduction

The current standard approach for the treatment of cervical disc disease is anterior cervical spinal fusion (ACDF) using the Smith-Robinson technique. This approach is considered relatively safe and effective; however, it has been reported to be associated with complications such as dysphagia, airway damage, and vocal disturbances in up to 79% of patients [1–3]. Although the exact pathophysiological mechanism of dysphagia after anterior cervical spine surgery is not fully understood, some studies have been proposed that it may be the effect of local soft-tissue edema, paralysis of the recurrent laryngeal nerve, or prevertebral soft-tissue swelling (PSTS) [4–6]. Severe dysphagia after ACDF has been demonstrated to lead to malnutrition, aspiration, increased risk of pulmonary complications, and increased medical costs [7].

To reduce the incidence and severity of dysphagia, steroids are often used in clinical practice. Corticosteroids are known anti-inflammatory agents that inhibit the production of inflammatory prostaglandins and cytokines. This inflammatory process is responsible for the swelling of the soft tissues, which eventually leads to compression of the esophagus and trachea. Several studies have shown that the use of systemic corticosteroids after anterior cervical spine surgery is beneficial in reducing the incidence and severity of dysphagia [8–10]. While these results are encouraging, systemic administration of corticosteroids is also associated with systemic side effects; this may be a limitation of this route of administration. For this reason, the efficacy of topical application of corticosteroids to the retropharyngeal space for anterior cervical fusion has been investigated. However, the reported results are not the same [11, 12], and the association between local application of corticosteroids on postoperative tissue swelling and dysphagia has not been well described. Therefore, to provide clear and uniform conclusions using the best available evidence, a systematic review and meta-analysis of randomized controlled trials was conducted. The primary and secondary objectives of this study were to determine whether the intraoperative application of topical corticosteroids in anterior cervical surgery has substantial clinical benefit in reducing the severity of dysphagia and identify any clinically relevant complications associated with corticosteroid therapy.

## 2. Materials and Methods

The study was conducted following the guidelines outlined in the PRISMA (preferred reporting items for systematic reviews and meta-analysis) statement [13]. The data included in this study are from published studies and are not directly relevant to the patients. Therefore, ethics committee approval and informed consent were not required.

### 2.1. Identification of Studies

A comprehensive literature search was conducted, to identify all published studies evaluating the efficacy of topically applied corticosteroids for anterior cervical fusion. Two independent reviewers conducted a systematic electronic search of PubMed, MEDLINE, EMBASE, the Cochrane Library, and Google Scholar. The search date is from the beginning to June 31, 2021. The keywords used for the search are “Dysphagia,” “Deglutition Disorders,” “Swallowing Disorders,” “Steroids,” “Cervical Vertebrae,” “Anterior Cervical Discectomy and Fusion,” “ACDF” and their near synonyms, etc. There are no restrictions based on publication date, language, or follow-up.

### 2.2. Assessment of Eligibility

The following criteria were used for study inclusion: (1) adult patients with cervical spondylosis undergoing single- or multilevel anterior decompression-only fusion; (2) perioperative topical corticosteroids, placebo in the control group, and no other interventions for dysphagia; and (3) RCT design.

The exclusion criteria are as follows: (1) trials that explicitly include cases of trauma, tumor, or infection; (2) abstracts, letters, or conference proceedings; and (3) studies for which no extractable data are available.

### 2.3. Selection of Literature

It was filtered by scanning the titles of each study to filter out inappropriate articles. An independent reviewer then reviewed the abstracts of the remaining studies and selected those that were potentially relevant to our study. Two authors independently reviewed the full text of these articles as well as the references for additional research. We then critically evaluated the studies according to the inclusion and exclusion criteria and assessed the quality of the randomized controlled trials according to the suggested checklist. All differences were resolved by consensus through discussion and further confirmed by a third author.

### 2.4. Assessment of Risk of Bias and Data Extraction

Study quality was assessed using version 2 of the Cochrane Risk of Bias Tool for Randomized Trials (RoB 2). Two reviewers applied all criteria to each study independently, using a uniform methodology. Clinical outcome data from individual studies were independently extracted into spreadsheets by 2 reviewers and reviewed against the original information to avoid errors. Data extracted from the study included identifiers, study characteristics, patient demographics, interventions, surgical data, perioperative PSTS, clinical dysphagia outcomes, visual analog scale (VAS), and complications, and disagreements were resolved through discussion and negotiation. PSTS is measured as the ratio of the measured anterior soft-tissue thickness of the vertebral body to the anterior-posterior (AP) diameter of each vertebral body. To compare the overall PSTS of the two groups, the mean PSTS at C3, 4, and 5 where edema was observed to be most pronounced was defined as the prevertebral soft-tissue swelling index (PSTSI). PSTSI eliminates interindividual differences, such as vertebral width or any other abnormalities in the cervical or retropharyngeal space, facilitating comparisons with other postoperative patients as well as with the patients themselves [14, 15].

### 2.5. Statistical Analysis

The meta-analysis was analyzed using Review Manager 5.3 (RevMan 5.3. Ink, Cochrane Collaboration, Oxford, United Kingdom). The *I*^2^ statistic (ranging from 0 to 100%) was used to quantify the heterogeneity between studies. *I*^2^ values >50% indicate significant heterogeneity, and random effects analysis was used to compare heterogeneous results. There is likely to be a high degree of heterogeneity between different randomized controlled trials due to clinical and methodological factors. Thus, even if *I*^2^ is small, the random effects model is applicable to the entire meta-analysis. Continuous variables are reported as mean differences and 95% confidence intervals, such as time to surgery, and dichotomous variables (e.g., complications) are reported as risk ratios and 95% confidence intervals; *P* value < 0.05 was considered to indicate a significant difference. Funnel plots were performed to assess publication bias.

## 3. Results

### 3.1. Search Results

**Figure 1:**
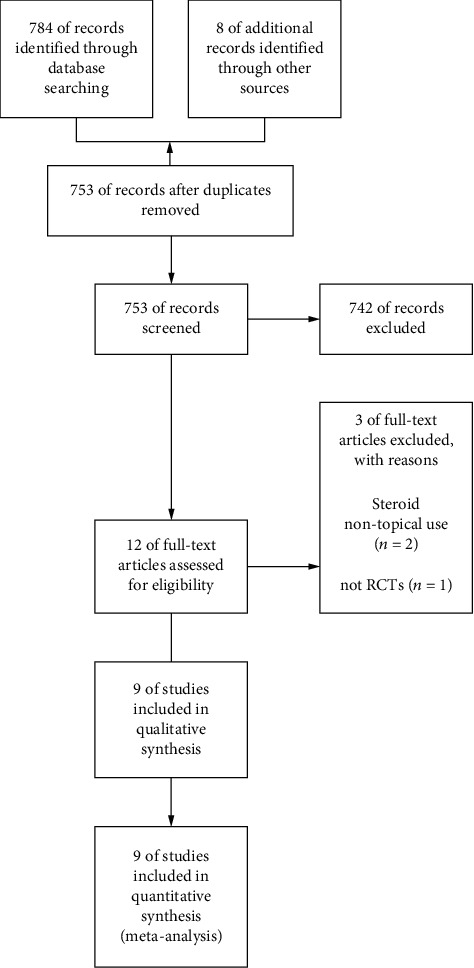
PRISMA flowchart for the literature search.

The flowchart of the search is shown in Figure 1. The search yielded 792 studies, of which 39 were duplicates. 742 studies were excluded based on title, abstract, and full-text screening, leaving 12 possible articles. Three other studies were excluded after full-text review because they did not use topical steroids or were not RCTs. Of the nine articles included, all were eligible for the meta-analysis. These studies included a total of 632 patients, of which 327 constituted the topical steroid group and the placebo group consisted of the remaining 305 patients.  Table 1 summarizes the characteristics of the eight included articles.

### 3.2. Risk of Bias

**Figure 2:**
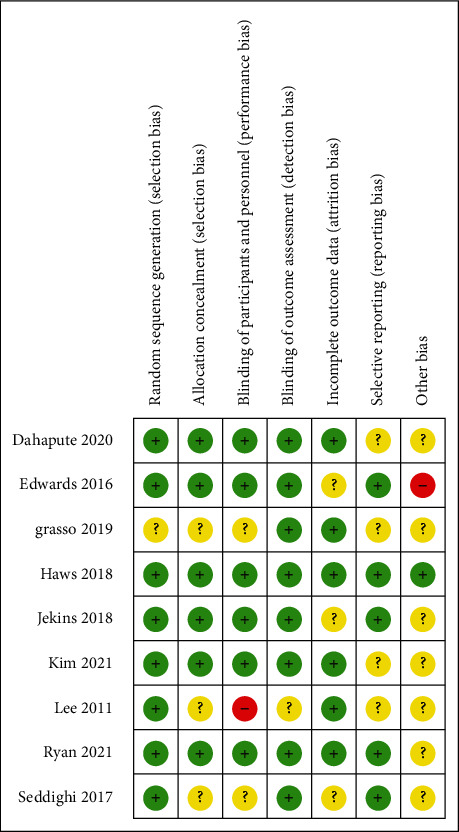
Risk-of-bias assessment.

The Cochrane risk of bias assessment for RCTs is shown in Figure 2. Two studies were found to have a “high” risk of bias, primarily attributable to the randomization process and outcome measures. For all studies, we used the modified Jadad scale to evaluate the qualities of them, where 3-5 scores mean high quality and 0-2 scores mean low quality. For quality assessment, all studies included in our research were of high quality [22] (Table 2).

### 3.3. Primary Outcome

**Figure 3:**
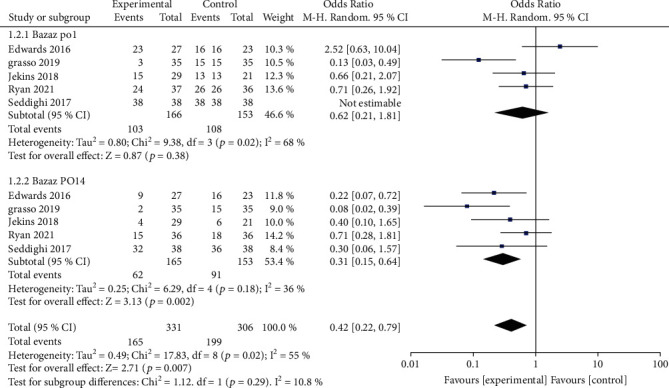
Primary outcome on the first postoperative day. Bazaz assessed no significant difference in the incidence and severity of dysphagia between the two groups. However, the steroid group was significantly lower than the control group at 14 days postoperatively. PO: postoperative.

**Figure 4:**
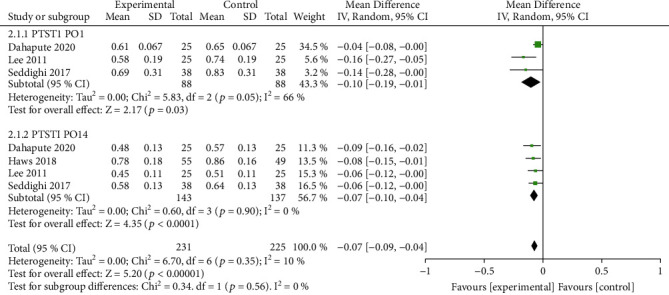
PSTSI was significantly different in the steroid group on the first postoperative day and on postoperative day 14.

In this series, the most commonly used tool for assessing dysphagia is the Bazaz scale [20, 21, 23] or its modified version, the Modified Dysphagia Scoring System (MDSS) [18, 19]. The Bazaz scale and MDSS have 4 levels: none, mild, moderate, and severe. Meta-analysis of the Bazaz scale of dysphagia showed no significant difference on the postoperative day (*P* = 0.38); however, at 2 weeks postoperatively, the overall incidence and severity of dysphagia were significantly lower in the patients in the topical steroid group than in the control group (*P* = 0.002) (Figure 3). Meta-analysis showed statistically significantly lower PSTSI in the topical steroid group than in the control group both one day after surgery and 2 weeks after surgery (1 day, *P* = 0.03 and 2 weeks, *P* < 0.0001) (Figure 4).

### 3.4. Secondary Outcome

**Figure 5:**
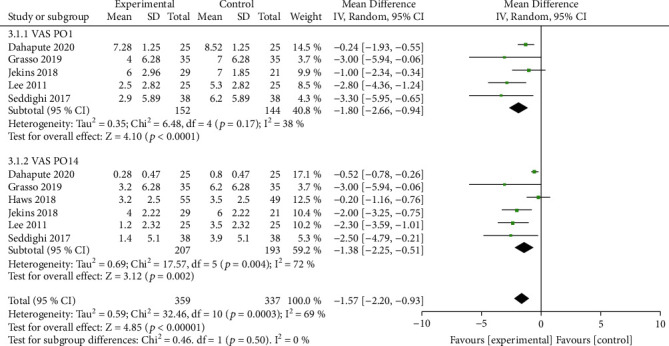
Secondary outcome VAS scores was significantly lower in the steroid group than in the control group on both postoperative day 1 and day 14.

A total of five studies reported VAS scores at the follow-up time points. VAS scores were significantly lower in the topical steroid group than in the control group one day postoperatively (*I*^2^ = 38%, *P* < 0.0001) versus 2 weeks postoperatively (*I*^2^ = 72%, *P* = 0.002) (Figure 5).

### 3.5. Publication Bias and Sensitivity Analysis

**Figure 6:**
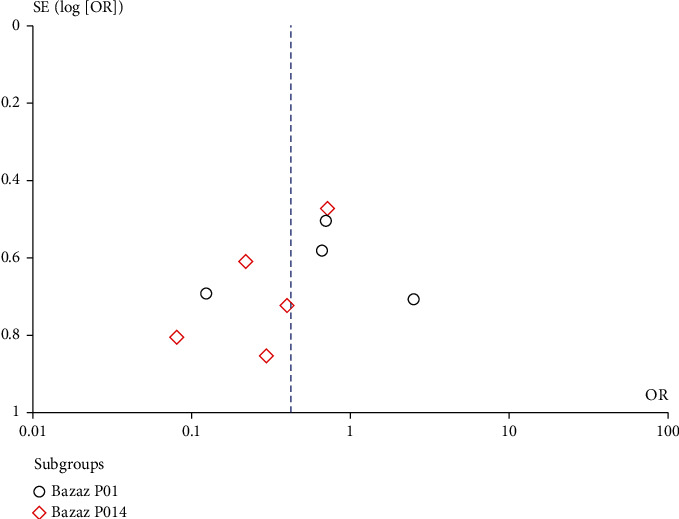
Funnel plot of the Bazaz scores.

**Figure 7:**
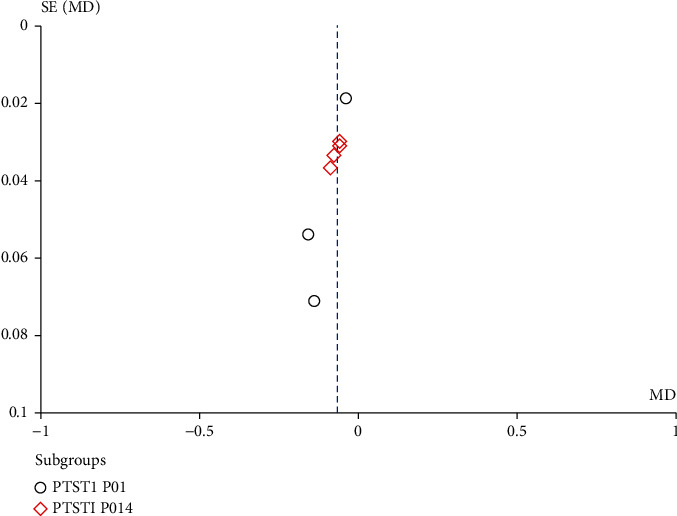
Funnel plot of the PSTSI.

We performed funnel plotting of the postoperative Bazaz score as well as the PSTSI, and the funnel plot shows a symmetric distribution (Figures 6 and 7). Although the statistical power was limited by the total number of studies, no significant bias was found.

To determine the effect of each study on postoperative Bazaz scores, postoperative PSTSI, and VAS scores, we perform a sensitivity analysis to verify the robustness of our results. No significant effect on the results was observed after excluding any single study, suggesting that the results of this meta-analysis are relatively robust.

## 4. Discussion

ACDF is an effective treatment for degenerative cervical spine disease when nonsurgical treatment has failed. Despite the clinical success of ACDF, postoperative problems may arise that the most common is dysphagia, the prevalence of which can be as high as 79%. Our meta-analysis of 632 patients in eight randomized trials found that topical steroids significantly reduced the occurrence and severity of dysphagia and improved neck pain after ACDF surgery. This is similar to the results of two previous systematic reviews and a meta-analysis [24–26]; they support the use of steroids to prevent dysphagia in patients undergoing anterior cervical fusion surgery. However, they included studies that also included systemic intravenous application of steroid hormones. Considering that topically applied steroids have a lower risk of systemic reactions or complications, our study compared the efficacy of topical steroid application with placebo control only. However, on the first postoperative day, there was no significant difference in the incidence of dysphagia between the two groups of patients. It is likely that the initial dysphagia on the first postoperative day is more of a mechanistic effect caused by the inherent manipulation of the esophagus by surgery [19], rather than due to swelling or inflammation, as the swelling of the anterior neck tissue gradually increases in the days following the procedure. And the topical application of steroids is a combination of steroids with gelatin sponges, which may delay the distribution and action of the drug, thus preventing any apparent effect on the first postoperative day [17]. There are no studies examining the exact pharmacokinetics of this route and method of administration [16].

The prevertebral soft tissues observed on a lateral radiograph consist of the muscles, ligaments, and cervical fascia of the pharynx or esophagus. Dissecting and stretching them during ACDF, edematous and inflammatory changes occur which leads to muscle and subperiosteal hemorrhage and soft-tissue swelling, resulting in the development of PSTS [27]. Dysphagia is common after ACDF surgery, and several studies have shown that the occurrence and severity of postoperative dysphagia are related to the degree of PSTS. Related reports indicate that the most severe PSTS occurs around the C4 vertebral body [14], and this is consistent with the findings of Suk et al. Therefore, the mean value of PSTS at C3, 4, and 5 (PSTSI) was used as the standardized comparison parameter. This meta-analysis found that topical steroid hormone application reduced the degree of PSTSI in patients on the first postoperative day, and at the second postoperative week, the PSTSI values were significantly lower in the steroid group compared to the control group. The incidence of painful dysphagia immediately after surgery was significantly lower in the steroid group than in the control group, and recovery was also faster; this is attributed to the control of the local inflammatory response by steroids and the subsequent reduction of the PSTS effect.

Esophageal perforation, one of the most dreaded complications of ACDF, has an incidence of 0.02% to 1.52% [28]. Topical steroids, on the other hand, increase the chance of perforation because they may reduce the ability of soft tissues to heal on their own. Only one study in this analysis reported 2 cases of delayed esophageal perforation [11]; they cautioned against the use of topical retropharyngeal steroids in patients with a long history of chronic steroid application. Corticosteroids reduce inflammation by decreasing neutrophil adhesion to the vascular endothelium and inhibit macrophages by limiting chemotaxis, phagocytosis, and cytokine release. However, while steroids suppress inflammation, they also increase the risk of infection. Dahapute et al.'s study showed that there were no significant differences in postoperative inflammatory indicators of white blood cell count and CPR between the two groups, and one case of infection was reported in the placebo group. Corticosteroids can promote apoptosis of osteoblasts and bone cells, which may affect bone healing as well as increase the risk of prosthetic joints. In this meta-analysis, although some studies reported cases of postoperative pseudarthrosis, but at subsequent follow-up, there were signs of bone nonunion in both groups, while excluding the possibility of a significant effect of steroids on bone healing [15]. However, this result should be interpreted with caution, because two of the eight studies did not report infection rates and four did not report pseudarthrosis rates. It is possible that the distant adverse effects and complications of steroids were not captured due to the short follow-up period.

The current study observed some limitations. Given the variation in the types and doses of steroids used in each study, we are unable to provide information on the best treatment regimen for reducing the rate and severity of postoperative dysphagia after ACDF, and better treatment doses may exist. We suggest that a relevant randomized controlled trial could be conducted to find the best clinical treatment option to reduce postoperative dysphagia using different types and different doses of topical steroids. Currently, one of the challenges in evaluating dysphagia is that there is no “gold standard” outcome indicator. Therefore, the included studies took different outcome scores for the postoperative dysphagia measure, so comparable data were not available for some of the studies, which may have biased the results. Also, differences in the design, definition, and outcome measures of studies of postoperative dysphagia in ACDF may be one reason for the wide variation in reported prevalence. Although relevant patient-centered dysphagia questionnaires and indicators have been validated, there is a subjective component to patient response, and more invasive modalities such as barium swallow tests and video laryngeal endoscopy can eliminate this subjective factor. In addition, the greatest change in dysphagia severity in the included study population appeared to occur in the first 2 weeks postoperatively. However, incidence and severity were measured only a small number of times during this period, thus obscuring the exact onset and duration of postoperative dysphagia that may be reduced by topical steroids in patients undergoing ACDF surgery. In the included studies, the short- to medium-term follow-up may have missed the time to maximum benefit for patients, as well as long-term outcome indicators, including fusion rates. However, we can say with confidence that the use of topically applied steroids is effective and does not significantly increase the risk of complications. But the differences between the included studies make it difficult to provide clear and specific recommendations. Therefore, future studies could include nonsubjective measures of dysphagia, more frequent documentation of patient-reported dysphagia, larger study populations, subgroup analyses, and cost-effectiveness analyses of intraoperative steroid use to reduce dysphagia after ACDF surgery.

## 5. Conclusion

This meta-analysis found moderate-quality evidence supporting perioperative topical steroid administration as an adjunct to anterior cervical spine surgery to reduce the incidence and severity of dysphagia compared with placebo. Future high-quality randomized comparative effectiveness trials are assured.

## Figures and Tables

**Table 1:** Baseline characteristics of studies included in the systematic review.

Study (year)	Experimental data			Control data				
	Patients	Mean age (yr)	Male (%)	Patients	Mean age (yr)	Male (%)	Outcomes recorded	Follow-up (month)
Dahapute 2020 [15]	25	50.4	76	25	50.4	76	PSTS, VAS, NDI	12
Ryan 2021 [16]	37	59	41	36	57	50	Bazaz, EAT-10	3
Kim 2021 [17]	56	58.1	48.2	53	58.4	54.7	EAT-10, SWAL-QOL, VAS, NDI	1
Haws 2018 [12]	55	49.4	56.4	49	50.6	61.2	PSTS, SWAL-QOL, VAS	3
Jekins 2018 [18]	29	55.6	51.7	21	54	52.3	Bazaz, EAT-10, VAS neck pain, NDI	12
Edwards 2016 [19]	27	54	41	23	54.5	39	Modified Dysphagia Scoring System	1
Lee 2011 [11]	25	54.3	72	25	50.9	56	PSTS, VAS, NDI	22
Seddighi 2017 [20]	38	49.3	47.3	38	50.2	42.1	Bazaz, PSTS, VAS	6
Grasso 2019 [21]	35	46.1	51.4	35	45.5	48.5	Bazaz, VAS	12

**Table 2:** Results of equality evaluation of included trials.

Study (year)	Random sequence generation	Allocation concealment	Blindness	Withdrawal and lost visits	Modified Jadad scale
Dahapute 2020	Random grouping	Appropriate	Appropriate	Describe	7
Ryan 2021	Random grouping	Appropriate	Appropriate	Describe	7
Kim 2021	Random grouping	Appropriate	Appropriate	Describe	7
Haws 2018	Random grouping	Appropriate	Appropriate	Describe	7
Jekins 2018	Random grouping	Appropriate	Appropriate	Unclear	6
Edwards 2016	Random grouping	Appropriate	Appropriate	Unclear	6
Lee 2011	Random grouping	Unclear	Unclear	Describe	5
Seddighi 2017	Random grouping	Unclear	Unclear	Unclear	4
Grasso 2019	Random grouping	Unclear	Unclear	Unclear	4

## Data Availability

The data supporting this meta-analysis are from previously reported studies and datasets, which have been cited. The processed data are available from the corresponding author upon request.

## Abbreviations

ACDF: Anterior cervical spinal fusion
PSTS: Prevertebral soft-tissue swelling
PSTSI: Prevertebral soft-tissue swelling index
MDSS: Modified Dysphagia Scoring System.
